# Generalisable Overview of Study Risk for Lead Investigators Needing Guidance (GOSLING): A data governance risk tool

**DOI:** 10.1371/journal.pone.0309308

**Published:** 2024-08-20

**Authors:** Anmol Arora, Adam Loveday, Sarah Burge, Amy Gosling, Ari Ercole, Sarah Pountain, Helen Street, Stephanie Kabare, Raj Jena

**Affiliations:** 1 School of Clinical Medicine, University of Cambridge, Cambridge, United Kingdom; 2 Cambridge University Hospitals NHS Foundation Trust, Cambridge, United Kingdom; 3 Department of Oncology, Cancer Research UK Cambridge Centre, University of Cambridge, Cambridge, United Kingdom; 4 University Hospitals Birmingham NHS Foundation Trust, Birmingham, United Kingdom; 5 Department of Oncology, University of Cambridge, Cambridge, United Kingdom; Fundación Universitaria del Área Andina, COLOMBIA

## Abstract

**Introduction:**

Digitisation of patient records, coupled with a moral imperative to use routinely collected data for research, necessitate effective data governance that both facilitates evidence-based research and minimises associated risks. The Generalisable Overview of Study Risk for Lead Investigators Needing Guidance (GOSLING) provides the first quantitative risk-measure for assessing the data-related risks of clinical research projects.

**Methods:**

GOSLING employs a self-assessment designed to standardise risk assessment, considering various domains, including data type, security measures, and public co-production. The tool categorises projects into low, medium, and high-risk tiers based on a scoring system developed with the input of patient and public members. It was validated using both real and synthesised project proposals to ensure its effectiveness at triaging the risk of requests for health data.

**Results:**

The tool effectively distinguished between fifteen low, medium, and high-risk projects in testing, aligning with subjective expert assessments. An interactive interface and an open-access policy for the tool encourage researchers to self-evaluate and mitigate risks prior to submission for data governance review. Initial testing demonstrated its potential to streamline the review process by identifying projects that may require less scrutiny or those that pose significant risks.

**Discussion:**

GOSLING represents the first quantitative approach to measuring study risk, answering calls for standardised risk assessments in using health data for research. Its implementation could contribute to advancing ethical data use, enhancing research transparency, and promoting public trust. Future work will focus on expanding its applicability and exploring its impact on research efficiency and data governance practices.

## Introduction

The expansion of digital health records and biomedical datasets, coupled with the development of novel analytical techniques such as machine learning, presents an unprecedented opportunity for clinical research. However, digitisation of patient data also introduces complex challenges in data governance, privacy, and ethical considerations. Digital records can be easily duplicated and transmitted across platforms and borders, increasing the risk of unauthorised access and complicating the enforcement of consistent privacy standards. As the healthcare sector navigates the delicate balance between leveraging data for innovation and safeguarding patient privacy, the need for effective data governance tools has become increasingly apparent. The use of deidentified routinely collected data in healthcare research has become increasingly significant, offering substantial benefits for public health insights, policy making, and personalised medicine development. De-identification involves removing or modifying personal information from health records to protect individuals’ privacy, allowing researchers to access valuable datasets without compromising patient confidentiality. The distinction between de-identified and anonymised data is subtle, whereby de-identification involves masking identifiers to prevent identification without additional information, whereas anonymisation would irreversibly detach potentially identifiable labels from the data, so that data cannot be linked back to an individual, even with additional information.

Access to routinely collected medical data for clinical research requires a valid, legal basis. In the absence of explicit consent from patients, the legal basis commonly used for health research by academic institutions is for a *“public task”* [1]. Special category data may be processed under the condition of *“Archiving*, *research and statistics”* [2]. This access to routinely collected medical data relies upon the fundamental assumption that the processing of personal health information in research and development will improve patient care. The NHS England Constitution promises to use de-identified routinely collected data for this purpose [3]. It is well-documented that risk aversion and complicated data governance processes in the UK may act as a barrier to research and development that relies upon secondary usage of routinely collected health data [4].

Risks associated with usage of health data for research can broadly be categorized into three main types: legal, governance, and reputational risks. Legal risk pertains to the potential for legal consequences arising from data use, such as breaches of data protection laws. Governance risk involves the potential for non-compliance with institutional policies and guidelines. Reputational risk relates to the potential damage to the institution’s or researchers’ reputation due to perceived mishandling or misuse of data. This can affect public trust and the willingness of individuals to participate in future research. In reality, there is considerable overlap between these as “risky” practices will likely have legal, governance and reputational implications.

Whilst there is a wealth of literature offering core principles for the function of data access committees and overarching advice, this study presents the first open-access framework for assessing the data-related risks of a project [5,6]. Although there has been previous work seeking to audit compliance with privacy, data governance and ethical principles at an institutional level, such as through the Privacy and Ethics Impact and Performance Assessment (PEIPA), there is still no consensus method of assessing whether specific projects are suitable for accessing routinely collected health data [7]. In the United Kingdom, there have been calls for increased resources to aid with data governance approvals as the current process can be unclear, convoluted and burdensome [4]. Furthermore, data governance processes are widely heterogeneous between countries and differ between institutions, even when common principles are being followed [8].

This study combines guidance from General Data Protection Regulation (GDPR), Information Commissioner’s Office, General Medical Council, Department for Digital, Culture, Media and Sport (DCMS), Five Safes, Health Insurance Portability and Accountability Act (HIPAA), Health Research Authority and the National Health Service [9–16]. Experts in data governance from Cambridge University Hospitals, University Hospitals Birmingham and the Cancer Research UK Cambridge Centre also inputted on the design of the tool, alongside members of the public. The tool focusses on legal, governance and reputational risk factors, rather than ethical factors. The tool is not designed to replace data governance committee reviews, but rather to be used as a triage system, to identify low and high-risk projects. Projects which are flagged by the tool as low-risk may require little scrutiny by a data access committee whereas those which are high-risk must be interrogated further. By releasing the tool in an open-access fashion with full transparency of scoring weights for each question, researchers will be able to self-assess their project and make changes to reduce the risk score before submission. Although the tool is released with a set of pre-determined questions, model weights and score thresholds, individual institutions will be able to modify these if required.

## Methods

### Risk tool design and scoring

The scoring self-assessment was designed to minimise the number of free-text questions and, where possible, force users to select an option. The questions were grouped under 11 domains:

1. Project details (not used for scoring)
2. Eligibility and Data Usage (not used for scoring)
3. Types of data being requested
4. Special category data requests
5. Data sharing partners
6. Data access requirements
7. Security requirements for data storage
8. Data transfer between devices
9. Public involvement and engagement
10. Data transfer agreements
11. Further free-text information (not used for scoring)

Each question was assigned a weight which would either increase (higher risk) or decrease (lower risk) the cumulative data risk score by a set multiplier depending on the response. A starting total score of 50 was set to allow for reductions in score. Scoring weights were determined by consensus between the study group, with input from patient and public members. The use of free-text questions was minimised as it would be infeasible for these to contribute to scoring in an automated fashion.

The risk tool was tested on a sample of 15 studies, which included real and synthesised data request proposals. Real proposals were sourced from Cambridge University Hospitals, University Hospitals Birmingham and the CRUK Cambridge Centre. Including synthesised examples was particularly important to test the effectiveness of the tool at identifying very high-risk projects, which are uncommonly received in the real-world. Scoring weights for individual questions were refined to ensure that the tool was able to differentiate between low, medium and high-risk proposals. Thresholds for low, medium and high risk interpretations were agreed by consensus by the study team based on the results of the testing.

This study did not include research involving human participants, tissue, animals or plants.

### Public and patient involvement and engagement

Public and patient involvement and engagement (PPIE) was facilitated by the National Institute for Health and Care Research (NIHR) Cambridge Biomedical Research Centre (BRC). Nine public members, selected by the NIHR Cambridge BRC, anonymously provided feedback on the study, data risk tool and score weights for each question. All suggestions were implemented and communicated to the PPIE members, with no further revisions requested.

## Results

Table 1 includes the questions and scoring weights in the final GOSLING model. An interactive spreadsheet, which automates scoring, is included as Supplementary File 1. A publicly available interface is accessible at https://datarisktool.shinyapps.io/RiskScore/ and the code to generate or modify this is accessible at: https://github.com/anmolarora-98/datarisktool/ In the UK, hospitals are often affiliated with a local university in the form of teaching hospitals and the tool accounts for this.

**Table 1:** GOSLING data risk tool and scoring weights.

Question number	Question	Answer		Multiplier
1a	Title of Project			*x*
1b	Lead applicant			*x*
1c	Named co-applicants			*x*
1d	Please list any reference numbers, with dates, for existing project approvals, e.g. Research & Development, Integrated Research Application System (IRAS) or Research Ethics Committee. If none, please write N/A.			*x*
1e	Plain English summary (max 300 words)			*x*
1f	Scientific abstract (max 300 words)			*x*
1g	Conflicts of interest statement (including financial disclosures). Please include conflicts relevant to all co-applicants			*x*
1h	Which of the following best describes your study	Please select		*x*
1i	Please list the specific data that is being requested with a brief explanation of why this is required for the project			*x*
1k	Name of person completing form			*x*
2a	Do you require de-identified routinely collected patient data for your project?	Please select		*x*
2b	The project has ethical approval and patients consent to researchers accessing their routinely collected data for the purposes of this particular project	Please select		*x*
2c	The project has a favourable opinion from an NHS REC AND a section 251 consent waiver approval from the Confidentiality Advisory Group	Please select		*x*
2d	The project involves members of the hospital clinical care team who will access identifiable information in order to de-identify the data for analysis	Please select		*x*
2e	The project involves researchers who have (or will have) a research passport and EPIC access, but who are not members of the hospital team accessing *identifiable* information at any stage	Please select		*x*
3	Does your project involve any of the following types of data? Please select all that apply	Biometric data (e.g. fingerprints, retinal scans)	Please select	*1*.*25*
		Genomic data	Please select	*1*.*25*
		Linkage with data from outside *Host Organisation*	Please select	*1*.*25*
		DICOM or other imaging data which includes metadata e.g. CT/MRI scans, echocardiograms or cardiac MRIs with contextual clinical information or metadata	Please select	*1*.*25*
		Rare or unusual diagnosis either in general population or *Host Organisation* patients (criteria: UK general population prevalence <1 in 100,000 or <10 new diagnoses at *Host Organisation* per year)	Please select	*1*.*1*
		Rare or unusual medications or procedures at *Host Organisation* (<10 at *Host Organisation* per year)	Please select	*1*.*1*
		Outliers in the data set–age, weight, height, length of stay etc	Please select	*1*.*2*
		Digitised slides or other similar pathology data		*1*
		Specific timestamps (exact dates and times) relating to individuals, including date of death	Please select	*1*.*3*
		Photographs of the face or other identifying feature	Please select	*1*.*4*
		Free-text fields	Please select	*1*.*2*
4	Does your project involve any of the following types of data? Please select all that apply	Data relating to any of: race, ethnic origin, politics, religion, trade union membership, sex life, sexual orientation, domestic violence history, forensic history	Please select	*1*.*25*
		Particularly sensitive medical data such as HIV status or IVF treatment	Please select	*1*.*25*
		Data relating to children (under 18 years)	Please select	*1*.*2*
		Data relating to vulnerable adults, for example patients living in care homes or other institutions, or with learning difficulties or mental illness	Please select	*1*.*2*
4a	Where any of the options in question 4 have been selected, please justify inclusion of this data.			* *
5	How and where will data be used? Please select all that apply	Internal study within *Host Organisation* only	Please select	*0*.*9*
		Study involving transfer to *University Affiliated with Host Organisation*	Please select	*1*
		Study with an academic partner organisation in the UK	Please select	*1*.*05*
		Study with public or third sector organisation in the UK, including other NHS trusts	Please select	*1*.*05*
		Study with tech start up	Please select	*1*.*4*
		Study with established pharmaceutical company	Please select	*1*.*1*
		Study with established technology company	Please select	*1*.*1*
		Study with an organisation based outside the UK (including academic, public, commercial and third sector)	Please select	*1*.*2*
		Study entirely within a trusted research environment	Please select	*0*.*9*
		Other commercial organisation (will be reviewed separately)		*1*
5a	(If Applicable) For external organisations, where will the partner be processing the data:	In the UK	Please select	*1*
		Outside the UK but in the EEA	Please select	*1*.*05*
		Outside the EEA but in a country recognised with a GDPR adequacy decision	Please select	*1*.*05*
		Outside the EEA but in a country recognised with a GDPR partial adequacy decision (e.g. USA, Australia, Japan, Canada)	Please select	*1*.*1*
		Outside the EEA and in a country without GDPR adequacy decision	Please select	*10*
5b	Please name any organisations identified in Q5			* *
6	Who will have access to the de-identified data?	Data will only be accessed by members of the clinical care team at *Host Organisation*, other *Host Organisation* staff with a substantive contract or with a non-research contract	Please select	*0*.*9*
		Data will only be accessed by academic researchers (usually *University Affiliated with Host Organisation* or PPIE) with an honorary research contract or letter of access for *Host Organisation*	Please select	*0*.*95*
		In addition to or instead of the above options, data will be accessed by staff from another NHS partner organisation, with or without a research passport/honorary contract	Please select	*1*.*05*
		In addition to or instead of the above options, data will also be accessed by staff from another academic partner organisation, third sector organisation or PPIE members who do not fit into any other category. They may or may not have a research passport/honorary contract	Please select	*1*.*05*
		In addition to or instead of the above options, data will also be accessed by staff from a commercial partner organisation. They may or may not have a research passport/honorary contract	Please select	*1*.*1*
7	What are the security arrangements for data storage? Please select all that apply	Within the *Host Organisation* IT environment **ONLY**, including BYOD	Please select	*0*.*9*
		Within an area of the University of *University Affiliated with Host Organisation* covered by an NHS toolkit	Please select	*1*
		Within an area of the University of *University Affiliated with Host Organisation* not covered by an NHS toolkit	Please select	*1*.*05*
		Organisational secure electronic devices (laptops, tablets, smart phones)	Please select	*1*
		Personal electronic devices (laptops, tablets, smart phones)	Please select	*10*
		Encrypted mobile media (thumb drives, mobile hard drives, magnetic media)	Please select	*1*
		Encrypted cloud storage not covered above but ISO270001 compliant	Please select	*1*
		Paper records/hard copies subject to the trust policy on sensitive documents	Please select	*1*
		Other (detail):		* *
8	How will the data be transferred between devices?	Not applicable	Please select	*0*.*8*
		Secure File Transfer Protocol	Please select	*0*.*95*
		Encrypted cloud storage (ISO270001 compliant)	Please select	*1*
		Secure email server (e.g. *Host Organisation* email, NHS.net)	Please select	*1*
		Standard email	Please select	*10*
		Encrypted mobile media (thumb drives, mobile hard drives, magnetic media)	Please select	*1*.*05*
		Standard mobile media	Please select	*10*
9	Please indicate if any of the following apply to your project?	Patients and public have been involved in the design of this study, including membership on the research team or close involvement in the formulation of the study	Please select	*0*.*8*
		Patients and public have been consulted on the study but not directly involved in its design *[Do not select this option if the above option has already been selected]*	Please select	*0*.*9*
		There is a protocol for the study in the public domain, e.g. as a published article or on a public repository	Please select	*0*.*95*
		A plain-English summary will be made available online, aimed specifically for public consumption e.g. on a website with an avenue for members of the public to contact the team if needed for further information *[This does not include mandatory reporting on data use registries]*	Please select	*0*.*95*
10	Please indicate if any of the following apply to your project?	This study has received NHS or University ethics approval	Please select	*0*.*85*
		There will be a data transfer agreement (DTA) in place for this data transfer/exchange	Please select	*0*.*95*
		This study has received specific research funding after review by a funding body	Please select	*0*.*9*
		*Host Organisation* maintains control of the data retention period	Please select	*0*.*95*
		*Host Organisation* maintains control of data access	Please select	*0*.*95*
		If there is a relevant data transfer agreement (DTA) for this project, please list it here:		*x*
11a	Please describe the extent to which patients and the public have been involved in designing this study. Include information about how PPIE members were recruited, specific feedback and changes that have been made as a result of PPIE and plans for ongoing input (max 250 words)			*x*
11b	Please provide details of the ethics review for this project (max 250 words)			*x*
11c	Please provide details of the funding that has been provided for this project (max 250 words)			*x*
11d	If applicable, please attach the data protection impact assessment that has been completed specifically for this study (optional) (max 250 words)			*x*

The tool was piloted on 15 real and synthesised projects to review the face validity of the scoring and to ensure the questions were clear for researchers. These ranged from simple projects where data was contained within the host organisation to much riskier projects, involving special category data (such as genomic or biometric data) being shared with commercial partners. Novel research methodologies such as federated learning were also included in testing. Federated learning projects would be expected to fall in the ‘medium risk’ range, because it was expected that they should require discussion at a data governance meeting but that they are likely to be approved, providing that data is not leaving the host institution and if there are no concerning risk factors. A sample of results of the testing on nine diverse synthesised projects is shown in Table 2. Due to the sensitive nature of the real projects, the results are not being publicly published. Testing on real projects yielded comparable results, although there were few high-scoring outliers compared to the range of theoretical projects, which were created to assess whether GOSLING could pick up particularly high- and low-risk projects.

**Table 2:** A sample of nine synthesised projects, ranging from low- to high- risk, subjected to the GOSLING tool.

Synthesised project proposal summary	Subjective risk estimate	Specific risk or mitigating factors	GOSLING score
An established technology company with ethical approval seeking to receive multi-modal datasets to support an AI based risk stratification tool in Cardiac Disease	High	Multi-modal rare disease varied data including structured and unstructured datasets being shared with a secure environment outside the NHS and the UK but within the EU. Data being shared includes genomic, biometric and imaging data.	189.0
The paediatric department request genomic and microbiome data to be shared with a local medtech start-up for developing an algorithm capable of predicting risk of neurodegenerative diseases	High	Children’s genomic data being shared with a commercial medtech start-up with a data transfer agreement in place	109.7
A technology company looking to access Diabetic Retinopathy images in conjunction with associated patient data to help validate an algorithm that looks at assessing disease present in a patient’s eye	High	Datasets are curated and anonymised at NHS site. Anonymised datasets are made available in a secure internal Trusted Research Environment for analysis with oversight by the Trust.	88.2
Joint project between clinical researchers and a Canadian academic partner looking to combine databases of abdominal CTs from hospitals internationally with clinical outcome data in order to develop an algorithm that can pick up incidental findings of intussusception and assess clinical relevance in adult females	High	Collecting both imaging and clinical data for a small number of patients with a rare or unusual diagnosis and sharing this with an international partner. This is a funded study with a data transfer agreement are in place	85.1
Observational cohort study examining clinical presentation, diagnosis, and treatment of refractory and unexplained chronic cough	Medium	Imaging, free-text and specific timestamp data are being requested. Mitigated risk by those involved will hold contracts with organisation, with data agreement in place stating responsibilities and requirements of project. The Trust retains control over the data access and no data will leave the trust secure monitored environment.	66.4
An established pharmaceutical company is looking to access blood films from the hospital with limited associated clinical data to see if they can predict response to chemotherapy agents in myeloma	Medium	Sharing data with an established pharmaceutical company based in the UK using secure cloud storage, with a data transfer agreement in place	57.5
The Respiratory department seeks to collaborate with a consortium of UK-based NHS hospitals to produce a federated machine learning algorithm capable of diagnosing lung cancer from chest X-rays. No patient data will be shared with third-party organisations, only model weights from the local federated algorithm	Medium	Cloud-based storage will be required to run a federated model on the local centre’s data. The data, however, will not be shared with any parties outside the host institution. University ethics approval has been obtained	43.0
A risk prediction model using a large language model to predict the length of stay of a patient based only on the venous blood gas taken in the emergency department on first admission	Low	Specific timestamp data is requested. Data is only accessed by researchers based at the host institution, which remains in control of data access and the retention period. The study has received university ethics approval	29.1
The neurosurgical department within the host institution requests access to all data for all adult patients with extraventricular drains, looking to predict the risk of drain infection based on routine blood tests at the time of drain insertion	Low	Data is only accessed by researchers based at the host institution, which remains in control of data access and the retention period. The study has received university ethics approval	22.4

A consensus meeting was held on 26^th^ February 2024, within the study group, during which the performance of the tool during this pilot was reviewed. The study group collectively agreed upon a relative ranking of the projects based on risk and a subjective assessment of whether they were low, medium or high risk. They were not blinded to the GOSLING score. Based on the performance on the synthesised and real projects, the following scoring thresholds were determined for interpretation of the score:

- **0 to 30: Low-risk:** Very likely to be approved, unlikely to require in-depth review
- **31 to 69: Medium-risk:** May require additional information or review
- **Over 70: High-risk:** Requires a discussion with the Research and Development team prior to submission to the Data Governance Committee

## Discussion

The GOSLING data risk tool provides, to our knowledge, the first quantitative risk evaluation of access to routinely collected health data, confirming to overarching standards of proportionality and data minimisation. The tool supports the data minimisation principle as expressed in Article 5(1)(c) of the GDPR and Article 4(1)(c) of Regulation (EU) 2018/1725. The data minimisation principle dictates that the use of personal data must be *"adequate*, *relevant and limited to what is necessary in relation to the purposes for which they are processed*.*”* [17] Proportionality is a well-established principle within data governance, ensuring that the scrutiny of review that a proposal receives reflects the perceived risk [18]. In a proportionate data governance system, projects which are triaged as low-risk may be fast-tracked for approval with minimal scrutiny whilst those which are high-risk could theoretically still be approved, but must be carefully considered by a committee. As explained by McGrail et al (2015), this differs from a risk-minimisation approach, whereby data is only released if risks are essentially absent [18]. Central to the proportionate response is the understanding that data governance committees have a duty to share data appropriately as well as restrict data appropriately, as emphasised by the seventh Caldicott principle supported by the UK Government National Data Guardian for Health and Social Care, which suggests that the duty to share information can be just as important as the duty to protect confidentiality [19,20]. Therefore, as well as being able to screen for high-risk projects, the tool should also be capable of triaging low-risk projects, so that these can benefit from proportionate by a review committee.

The importance of this proportionate approach in reducing unnecessary impediments to low-risk projects has been highlighted in the Canadian Tri-Council Policy Statement on Ethical Conduct for Research Involving Humans (2018) [21]. One of the most comparable data risk assessments to the GOSLING tool is the data governance of the ScottisH Informatics Programme (SHIP), which provides a comprehensive framework to analyse the risk-level of a project based on the: public interest of the project, the data being requested, the researchers involved and the environment of the project. The GOSLING tool builds upon the SHIP model, providing a quantifiable summation of risk measures that can be used for initial triage [22].

**Fig 1:**
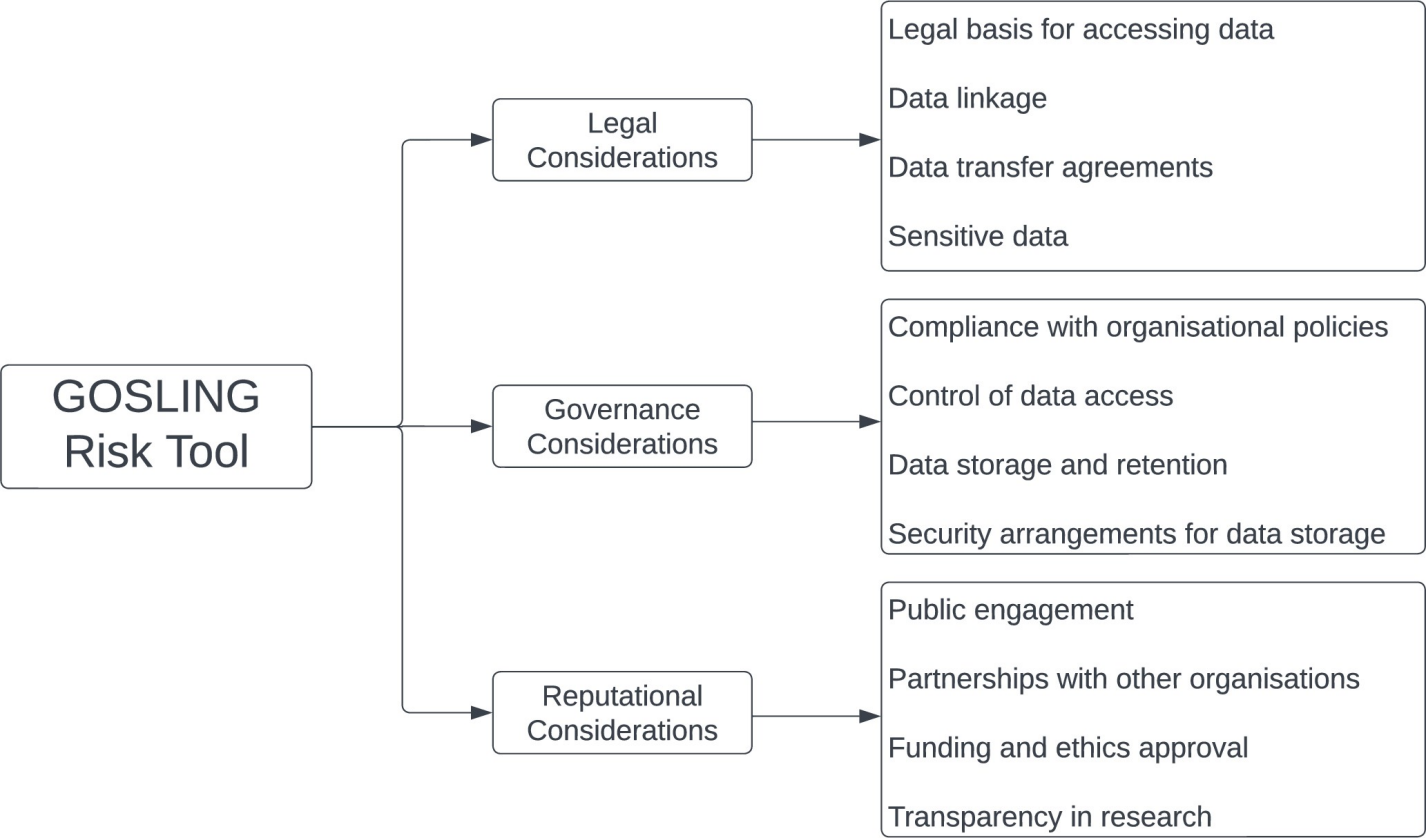
Data-related risk factors. High-level overview of data-related risk factors under the domains of legal, governance and reputational considerations. Note that in practice there is overlap between these three domains.

There are numerous frameworks that provide high-level guidance of principles that coordinating bodies should consider when appraising data access requests, however these usually involve a subjective review by a coordinating body on receipt of the application. The GOSLING tool adds to existing frameworks by allowing researchers to self-assess their risk and make necessary adjustments prior to submission. The tool considers legal, governance and reputational risk factors in order to generate an aggregate score (Fig 1). Questions 2 to 4 broadly consider the legality of the data access request, collating details on the legal basis to access the data. Questions 5 to 8 focus upon plans for the governance and handling of the data and these questions may be altered to suit institutional requirements. Information collected from questions 9 to 11 may affect institutional risk and these questions provide the opportunity for researchers to reduce their risk score such as by having incorporated public engagement. In practice, many questions can affect more than one type of ‘risk’, for example adherence to legal frameworks would likely breach institutional governance requirements and may carry associated reputational risk.

The need for a standardised risk-assessment tool for access to data is perhaps most eloquently described by the ‘Goldacre Review’, commissioned by the UK government in 2021 to assess how to improve the use of health data for research and analysis in the UK: “*The research and analytical community is extremely frustrated with the current arrangements around data access*. *Researchers and NHS service analysts can spend months or years trying to get multiple permissions from multiple parties…”* [23].

**Fig 2:**
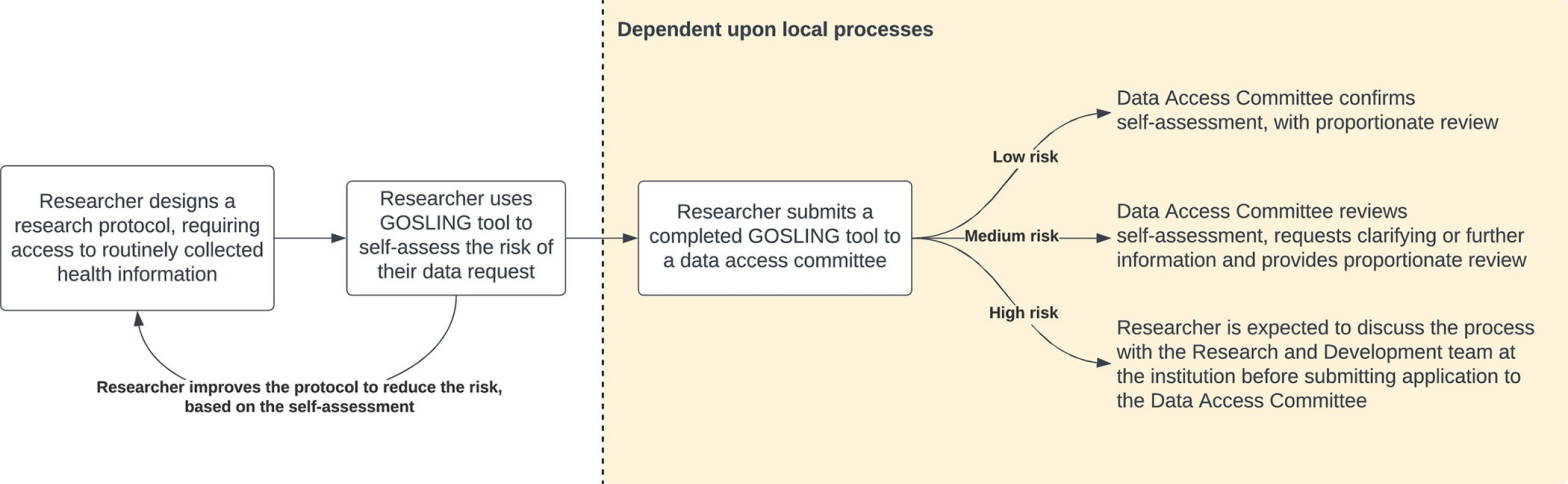
Example GOSLING implementation. Example schematic of how the GOSLING tool may be practically implemented to streamline the local processes of a data access committee.

As well as providing a framework for data governance committees to review requests, the tool serves the dual purpose of allowing researchers to self-appraise the risk of their requests (Fig 2). Previous tools have focussed on highlighting factors that increase the risk and drawing attention to these. This tool purposefully includes potentially ‘protective factors’ that reduce the risk score by enhancing transparency. If the study has been subjected to external scrutiny, either by the public or other research committees, this increases the likelihood that research activities align with public interest and therefore reduces reputational risk. These factors include:

- Patient and public involvement
- A public protocol and plain English summary
- Co-existent approvals for the project, including ethical approvals

By including protective factors, we highlight to researchers that it may be possible to reduce the risk of a high-risk project to an acceptable level without changing the study design, but instead by including protective factors. By releasing the tool, with all scoring weights, we encourage full transparency with the public about how routinely collected health data is being used. The inclusion of patient and public involvement as a protective factor serves an additional purpose of encouraging researchers to engage with this activity early on in their research process, strengthening their data access application. Public and public members have been consulted in the design of the tool, consistent with the principle of ‘participatory data governance’, a modern feature of data governance committees [24,25]. Members of data governance committees have also contributed to the design of the tool as co-authors of this manuscript and have been actively involved in its testing. In general, there was agreement between data access committee members and public members of the content of the tool. Public members suggested modifications to questions, which were incorporated. This both included suggesting new sources of risk that had not previously been considered (e.g. expanding the definition of sensitive data) and identifying areas of jargon that may not be easily interpretable by a non-specialist audience.

### Limitations

This data risk tool is specifically designed to appraise studies which are requesting data for routinely collected healthcare information, that do not have informed consent from patients. Where informed consent is obtained from patients the tool advises discussion with the governance team as there may be different legal and ethical processes for this. Although the tool could still be used for these consented data access requests, it is not intended for this purpose. The tool is intentionally designed not to appraise the worthiness of a research question but rather to focus on the data governance issues. In this way, the tool is not aimed towards use by funding bodies or ethics committees, who may focus on the utility of the research. The tool would not differentiate between two projects using the same data in the same manner for projects of varying utility. Although the tool incorporated guidance originating from outside the UK to inform the questions, it is UK-specific, referencing UK legislation and procedures. There is heterogeneity, even within the European Union, of processing of health data for research, including data linkage between databases [7]. The tool could be modified to suit a different region, but this should be done in consultation with data protection experts in the region. Further research is required to evidence what, if any, time saving is afforded to researchers and committees by using the tool. The tool has been tested and agreed upon by three institutions, but a follow-up study comparing the adoption by more institutions in the future would help to review how different institutions view data access requests and adjust scoring accordingly. The small sample size of the study is a limitation and the tool requires more testing on more projects. Details of real studies that have been evaluated with the tool have not been published as these were tested retrospectively and under the expectation that the details of the studies and authors would not be published publicly. Testing on real projects that have undergone data governance review would bias against the most high-risk projects, which are less likely to reach panel review without derisking. The synthesised project proposals helped to fill this gap and they were intentionally designed to capture a variety of risk profiles, ranging from low to high risk. The tool requires testing in a real-world environment, where researchers self-score applications with the tool and completed scoring proforma are reviewed by a data access committee. Testing to date has been in a controlled setting, applied to either synthesised studies or retrospective application to existing projects and resultantly the expert review of the projects by the study group has not been blinded to the performance of the tool. Importantly, for future testing of the tool it should be applied to projects in a variety of settings (e.g. primary care, secondary care and within research organisations) and efforts could be made to translate it for application in other languages or geographies to assist with external validation of its content.

### Future directions

This study represents, to our knowledge, the first publicly available tool for quantifying the relative risk of granting access to routinely collected health data for a project. This tool must not replace data access committees but rather it presents a mechanism for such committees to streamline the processing of data access requests and triage those which require most scrutiny. The tool might also be used by research governance teams within hospital trusts who triage and review requests, helping with the development of any data access or internal trust committees. We hope that its use may also lead to improved quality submissions of data access requests by allowing researchers to self-assess the risk of their project and showing them how risk mitigation measures may improve the likelihood of their access request being approved.

## Supporting information

S1 File Interactive GOSLING spreadsheet. An interactive offline version of the GOSLING tool in Microsoft Excel, which automates scoring.
(XLSX)

## References

[1] Using data about people in research. Accessed April 8, 2024. https://www.ukri.org/councils/mrc/facilities-and-resources/find-an-mrc-facility-or-resource/mrc-regulatory-support-centre/using-data-about-people-in-research/.

[2] What are the conditions for processing? Published December 18, 2023. Accessed April 8, 2024. https://ico.org.uk/for-organisations/uk-gdpr-guidance-and-resources/lawful-basis/special-category-data/what-are-the-conditions-for-processing/.

[3] The NHS Constitution for England. GOV.UK. Accessed February 6, 2024. https://www.gov.uk/government/publications/the-nhs-constitution-for-england/the-nhs-constitution-for-england.

[4] Jones MC, Stone T, Mason SM, Eames A, Franklin M. Navigating data governance associated with real-world data for public benefit: an overview in the UK and future considerations. *BMJ Open*. 2023;13(10):e069925. doi: 10.1136/bmjopen-2022-069925 37793928 PMC10551984

[5] Cheah PY, Piasecki J. Data Access Committees. *BMC Med Ethics*. 2020;21:12. doi: 10.1186/s12910-020-0453-z 32013947 PMC6998828

[6] Liyanage H, Liaw ST, Di Iorio CT, et al. Building a Privacy, Ethics, and Data Access Framework for Real World Computerised Medical Record System Data: A Delphi Study. *Yearb Med Inform*. 2016;(1):138–145. doi: 10.15265/IY-2016-035 27830242 PMC5171555

[7] Iorio CTD, Carinci F, Oderkirk J, et al. Assessing data protection and governance in health information systems: a novel methodology of Privacy and Ethics Impact and Performance Assessment (PEIPA). *J Med Ethics*. 2021;47(12):e23–e23. doi: 10.1136/medethics-2019-105948 32220868

[8] Torabi F, Squires E, Orton C, et al. A common framework for health data governance standards. *Nat Med*. 2024;30(1):26–29. doi: 10.1038/s41591-023-02686-w 38191614

[9] General Data Protection Regulation (GDPR) Compliance Guidelines. GDPR.eu. Accessed September 12, 2023. https://gdpr.eu/.

[10] Information Commissioner’s Office (ICO). Published March 1, 2024. Accessed March 3, 2024. https://ico.org.uk/.

[11] GMC. Using and disclosing patient information for secondary purposes. Published 2018. Accessed February 20, 2024. https://www.gmc-uk.org/professional-standards/professional-standards-for-doctors/confidentiality/using-and-disclosing-patient-information-for-secondary-purposes.

[12] Data Sharing Governance Framework. GOV.UK. Accessed March 3, 2024. https://www.gov.uk/government/publications/data-sharing-governance-framework/data-sharing-governance-framework.

[13] The ‘Five Safes’–Data Privacy at ONS | National Statistical. Accessed March 3, 2024. https://blog.ons.gov.uk/2017/01/27/the-five-safes-data-privacy-at-ons/.

[14] HIPAA Home | HHS.gov. Accessed March 3, 2024. https://www.hhs.gov/hipaa/index.html.

[15] Accessing data for research and analysis. NHS Transformation Directorate. Accessed March 3, 2024. https://transform.england.nhs.uk/key-tools-and-info/data-saves-lives/secure-data-environments/accessing-data-for-research-and-analysis/.

[16] Guidance for using patient data. Health Research Authority. Accessed March 3, 2024. https://www.hra.nhs.uk/covid-19-research/guidance-using-patient-data/.

[17] D | European Data Protection Supervisor. Published April 8, 2024. Accessed April 8, 2024. https://www.edps.europa.eu/data-protection/data-protection/glossary/den.

[18] McGrail KM, Gutteridge K, Meagher NL. Building on Principles: The Case for Comprehensive, Proportionate Governance of Data Access. In: Gkoulalas-Divanis A, Loukides G, eds. *Medical Data Privacy Handbook*. Springer International Publishing; 2015:737–764. doi: 10.1007/978-3-319-23633-9_28

[19] The Caldicott Principles. GOV.UK. Accessed March 20, 2024. https://www.gov.uk/government/publications/the-caldicott-principles.

[20] Information sharing and disclosure. UKCGC. Accessed March 20, 2024. https://www.ukcgc.uk/information-sharing-and-disclosure.

[21] Government of Canada IAP on RE. Tri-Council Policy Statement: Ethical Conduct for Research Involving Humans–TCPS 2 (2018). Published April 1, 2019. Accessed March 20, 2024. https://ethics.gc.ca/eng/policy-politique_tcps2-eptc2_2018.html.

[22] Laurie G, Sethi N. Information Governance of Use of Health-Related Data in Medical Research in Scotland: Towards a Good Governance Framework. *SSRN Electron J*. Published online 2012. doi: 10.2139/ssrn.2037117

[23] Goldacre B, Morley J. Better, Broader, Safer: Using health data for research and analysis. A review commissioned by the Secretary of State for Health and Social Care. *Dep Health Soc Care*. Published online 2022.

[24] de Freitas C, Amorim M, Machado H, et al. Public and patient involvement in health data governance (DATAGov): protocol of a people-centred, mixed-methods study on data use and sharing for rare diseases care and research. *BMJ Open*. 2021;11(3):e044289. doi: 10.1136/bmjopen-2020-044289 33722870 PMC7959217

[25] Kaye J, Terry SF, Juengst E, et al. Including all voices in international data-sharing governance. *Hum Genomics*. 2018;12:13. doi: 10.1186/s40246-018-0143-9 29514717 PMC5842530

